# Plant-Based COVID-19 Vaccines: Current Status, Design, and Development Strategies of Candidate Vaccines

**DOI:** 10.3390/vaccines9090992

**Published:** 2021-09-06

**Authors:** Puna Maya Maharjan, Sunghwa Choe

**Affiliations:** 1 G+FLAS Life Sciences, 123 Uiryodanji-gil, Osong-eup, Heungdeok-gu, Cheongju-si 28161, Korea; punamaya.maharjan@gflas.com; 2 G+FLAS Life Sciences, 38 Nakseongdae-ro, Gwanak-gu, Seoul 08790, Korea; 3 School of Biological Sciences, College of Natural Sciences, Seoul National University, Gwanak-gu, Seoul 08826, Korea

**Keywords:** COVID-19 vaccine, SARS-CoV-2, plant-based vaccine, VLP, subunit vaccine, clinical trial, *Nicotiana benthamaiana*, RBD, spike protein, glycoengineering

## Abstract

The prevalence of the coronavirus disease 2019 (COVID-19) pandemic in its second year has led to massive global human and economic losses. The high transmission rate and the emergence of diverse SARS-CoV-2 variants demand rapid and effective approaches to preventing the spread, diagnosing on time, and treating affected people. Several COVID-19 vaccines are being developed using different production systems, including plants, which promises the production of cheap, safe, stable, and effective vaccines. The potential of a plant-based system for rapid production at a commercial scale and for a quick response to an infectious disease outbreak has been demonstrated by the marketing of carrot-cell-produced taliglucerase alfa (Elelyso) for Gaucher disease and tobacco-produced monoclonal antibodies (ZMapp) for the 2014 Ebola outbreak. Currently, two plant-based COVID-19 vaccine candidates, coronavirus virus-like particle (CoVLP) and Kentucky Bioprocessing (KBP)-201, are in clinical trials, and many more are in the preclinical stage. Interim phase 2 clinical trial results have revealed the high safety and efficacy of the CoVLP vaccine, with 10 times more neutralizing antibody responses compared to those present in a convalescent patient’s plasma. The clinical trial of the CoVLP vaccine could be concluded by the end of 2021, and the vaccine could be available for public immunization thereafter. This review encapsulates the efforts made in plant-based COVID-19 vaccine development, the strategies and technologies implemented, and the progress accomplished in clinical trials and preclinical studies so far.

## 1. Introduction

Since the first report on the emergence of the severe acute respiratory syndrome coronavirus 2 (SARS-CoV-2) virus causing coronavirus disease 2019 (COVID-19) in China at the end of 2019, it has spread across 220 countries and territories, infected 160 million people, and caused the deaths of 3.3 million people as of June 19, 2021 [1]. The coalescence of advanced biotechnology and global scientists’ efforts made the COVID-19 vaccine available for public immunization within a year of SARS-CoV-2’s emergence, faster than ever in vaccine history. To date, 8 vaccines are approved for full use, 8 are authorized for limited use, 31 are in phase 3 clinical trials, 37 are in phase 2 clinical trials, 51 are in phase 1 clinical trials, and 185 are in preclinical studies [2,3]. All the potential vaccine production platforms have been exploited to develop a COVID-19 vaccine.

**Figure 1:**
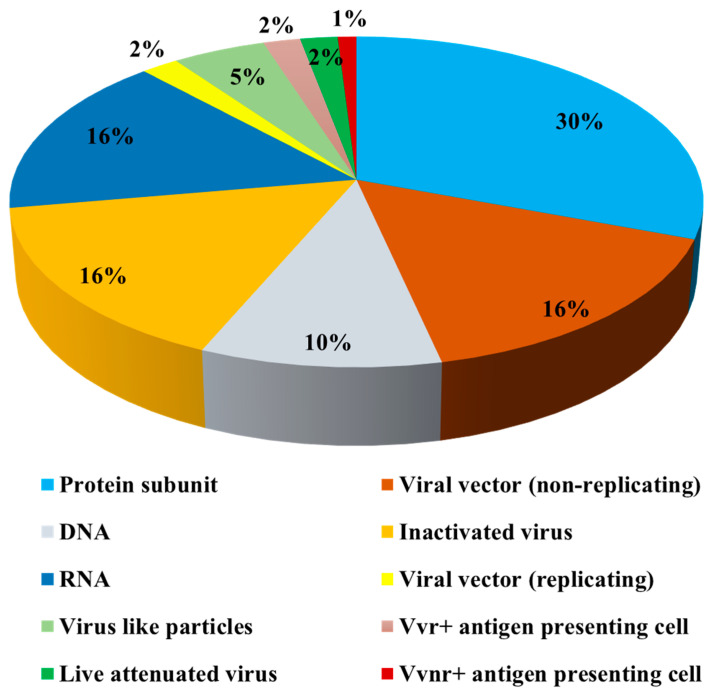
Vaccine platforms used to develop coronavirus disease 2019 (COVID-19) vaccine candidates. Data source: World Health Organization (WHO), 19 June 2021.

Egg-based vaccine production remains the most effective method for commercial production due to its excellent production capacity and low production cost [4]. However, the technology cannot be used for the COVID-19 pandemic because of the long production time and, most importantly, the inability of SARS-CoV-2 to replicate in hens’ egg cells [5]. Instead, many vaccine developers prefer advanced platforms, such as DNA, RNA, protein subunits, inactivated viruses, nonreplicating viral vector, virus-like particles (VLP), live attenuated viruses, inactivated virus, replicating viral vector (VVr), nonreplicating viral vector (VVnr), replicating viral vector with antigen-presenting cells, and nonreplicating viral vector with antigen-presenting cells [2,6,7]. Recombinant DNA technology-based production has dominated COVID-19 vaccine development, narrowing the whole virus vaccine to 18% of the total vaccines in clinical trials (Figure 1).

Plant-based production has received immense attention among vaccine researchers as a potential alternative to rapidly produce affordable COVID-19 vaccines and other therapeutics [8,9,10,11,12,13,14,15,16,17,18]. Plant-based production offers several advantages, such as low cost, rapidity, scalability, and safety. Moreover, plants can produce glycosylated recombinant proteins, which is not possible in an *Escherichia coli*-based culture system. As the glycosylation pattern affects the bioactivity of protein-based vaccines and therapeutics, recombinant proteins with a variable glycan pattern and improved efficacy can be generated by controlling glycosylation at different levels via glycoengineering of the host plant [19,20,21]. The technology has been used to produce a variety of pharmaceuticals, including vaccines, monoclonal antibodies, human growth hormones, immunomodulators, diagnostic reagents, and medical devices; some of them are in clinical trials [14,22,23,24,25,26]. Several correctly folded antigens against numerous infectious viruses, including influenza, Ebola, dengue, rotavirus, and norovirus, have been produced in plants and can induce neutralizing antibodies in both the animal model and humans [27,28,29,30,31].

The plant-based system is feasible to produce various types of vaccine, such as protein subunit, VLP, chimeric VLP (cVLP), and multiepitope vaccines, with implications of transient and stable expression [13]. Stable expression is suitable for producing vaccines that are continuously required in large quantities, because once the transgenic plant-expressing specific antigen is prepared, the product can be harvested for several generations, ensuring continuous manufacturing and availability [32]. In addition, when the transgenic system is applied to a host plant, such as rice, potato, or lettuce, an edible vaccine that induces humoral as well as cellular immune responses can be produced [33,34,35,36,37,38]. In contrast, the transient expression system can produce a target antigen within one week after introducing an antigen-coding sequence into plants, compared to one or several years via the transgenic expression system [18]. This unique feature of the transient expression system for rapid vaccine production tends to be the first and an excellent choice to produce a vaccine, especially in an emergency such as the COVID-19 pandemic. The plant transient expression system is much faster than any other system and produces a vaccine in 20 days after the protein amino acid sequence becomes available [39], with a yield of up to 4 g of green fluorescent protein (GFP)/kg fresh weight [40]. This enables the production of up to 10 million doses of the vaccine per month [41], making the system ideal for bulk production and for a quick response to an unexpected disease outbreak and pandemic.

The world’s first plant-produced vaccine was approved for Newcastle disease virus (NDV) by the US Department of Agriculture in 2006 for poultry [42,43]. The first plant-produced pharmaceutical approved by the US Food and Drug Administration (USFDA) for human use in 2012 was taliglucerase alfa enzyme (Elelyso), produced from genetically engineered carrot cells for treating Gaucher disease [27,44,45]. Researchers have attempted to develop a vaccine for COVID-19 by expressing SARS-CoV S1 protein in transgenic tobacco, tomato, and transplantomic lettuce [46,47]. Oral immunization of mice with S1 protein-expressing transgenic tomato induced a high level of SARS-CoV-specific immunoglobulin IgA, while parenteral immunization with tobacco produced S1-induced SARS-CoV-specific IgG in mice [46]. The plant-based recombinant protein production approach has been implemented for the development of vaccines, diagnostic reagents, and therapeutics for COVID-19 and has progressed into clinical trials [48,49,50,51,52,53,54,55,56,57]. The coronavirus virus-like particle (CoVLP) vaccine is in phase 3 and Kentucky Bioprocessing (KBP)-201 in phase 1/2 clinical trials, while IBIO-200, IBIO-201, IBIO-202, Baiya SARS-CoV vax 1, and many more are in the preclinical phase of development (Table 1) [51,58,59,60].

This review describes the recent progress in the development of plant-based COVID-19 vaccines and analyzes the research, design, and development strategies and the technologies employed so far by different biotech companies and research groups. Most importantly, it discusses recently available results of the clinical trials of plant-based COVID-19 vaccine candidates. The information provided in this review is based on the electronic data available in PubMed, Google Scholar, Google Search, clinical trials, different biotech companies’ websites, universities’ websites, and online news portals.

## 2. Plant-Based Vaccine Production for Epidemic Response

Plant-based vaccine production is a rapid, robust, scalable, and effective technology for virus outbreak response. In the 2014 Ebola outbreak, ZMapp, a cocktail of monoclonal antibodies produced in the tobacco plant, was administered to six affected people at a dose of 50 mg/kg body weight and resulted in the recovery of five patients [62,63]. The product was in the experimental phase at the time of the outbreak and received authorization by the USFDA for emergency compassionate approval for human use and clinical study in Africa [9,52]. This showcased the relevance of plant-based biopharmaceutical production technology and its speed, scalability, and efficacy to the world.

Plant-based vaccine production was prioritized as a potential rapid production technology to manufacture the influenza vaccine under the Blue Angel program, launched by the US Defense Advanced Research Projects Agency (DARPA), in order to accelerate vaccine production in response to the 2009 H1N1 influenza pandemic. In 2012, the program, in collaboration with Medicago, Canada, successfully manufactured 10 million doses of monovalent H1N1 VLP vaccine in tobacco plants within 30 days, accomplishing the proof-of-concept objective to demonstrate the potential scalability and rapidity of plant production to combat the influenza pandemic [41]. Numerous plant-based vaccines against a wide range of pathogens and diseases, including influenza, Ebola, rabies, hepatitis B, norovirus malaria, anthrax, and rotavirus, have entered different phases of clinical trials (Table 2) [27,36,48,50,58,59,62,64,65,66,67,68,69,70,71,72,73,74,75,76,77,78]. Recently, the quadrivalent vaccine developed by Medicago for seasonal influenza has completed a phase 3 clinical trial [48]. Advancements in plant-based pharmaceuticals in recent decades have strengthened the technology, and it is now a promising production system for fighting the COVID-19 pandemic. Currently, two plant-produced vaccines are in clinical trials and many others are in preclinical stages (Table 1).

Plant-based production has been used to develop vaccines against several epidemics. Vaccine development for the devastating plague pandemic was attempted by expressing F1 and V antigens in tobacco and tomato plants, which induced 50% protection in mice and 88% protection in nonhuman primates after a *Yersinia pestis* challenge (Table 3) [69,80,81,82]. Similarly, the tobacco-produced vaccine for yellow fever disease, targeting the envelope protein, elicited up to 100% of protection in mice and a cellular and humoral immune response in monkeys after pathogen challenge (Table 3) [82,83,84]. Numerous epitopes of the envelope glycoprotein and capsid proteins of human immunodeficiency virus (HIV) have been expressed in plants, such as lettuce, tobacco, arabidopsis, moss, and carrot, in order to develop a vaccine against acquired immunodeficiency disease, and they have been found to be effective in inducing a humoral and cellular immune response in mice (Table 3) [34,82,85,86]. In addition, plant-produced vaccines against dengue and Ebola have demonstrated the induction of antibodies in animal models [87,88,89].

## 3. CoVLP: A COVID-19 VLP Vaccine in a Phase 2/3 Clinical Trial

Of the five COVID-19 VLP vaccines in clinical trials listed by the World Health Organization (WHO), one plant-based VLP vaccine is in a phase 3 clinical trial. Medicago developed the CoVLP vaccine within 20 days after receiving the SARS-CoV-2 genetic sequence [39]. After encouraging positive results from phase 1 and completion of phase 2 studies, the company announced the beginning of a phase 3 clinical trial on 16 March 2021 [58].

To design a VLP, the company selected the spike protein (S protein) of SARS-CoV-2, the most widely used target antigen for COVID-19 vaccines among the structural envelope protein (E), membrane protein (M), and nucleocapsid protein (N) because of its high immunogenicity and crucial role in viral cell entry [90,91,92]. S protein is a surface glycoprotein that triggers the entry of the virus into the human cell via binding with the receptor angiotensin-converting enzyme 2 (ACE2), prevalent in human respiratory tract cells [92,93]. Most of the SARS-CoV-2-neutralizing antibodies from COVID-19-recovered patients were directed against the S protein [94,95,96]. Hence, the antibodies generated after vaccination with an S-protein-based vaccine can target the S protein to inhibit the natural infection by interfering with the binding of the virus and ACE2 during cell entry.

Medicago’s CoVLP vaccine mimics the surface structure of the natural SARS-CoV-2 virus with an antigenic moiety but is non-infectious and nonreplicating as it lacks the replicating viral RNA. Hence, the VLP is safe and can stimulate an immune response against the virus when administered to humans. The VLP is an advanced form of recombinant antigen, compatible and favorable for plant-based vaccine production. The VLP is also an improved alternative to recombinant antigens and is highly immunogenic because of its complex external structure similar to that of natural viral particles [97,98,99]. VLP-based vaccines share most of the properties of the natural virus, such as a repetitive surface geometry, a particulate nature, and the ability to stimulate innate and adaptive immune responses, but without the ability to replicate, making the VLP a protective and safe mode of vaccination [29,100,101,102,103,104,105]. Moreover, the manufacturing of highly infectious pandemic virus particles requires high-level safety measurement; however, VLP production does not require any costly safety measurement since VLPs are composed of only non-infectious recombinant proteins. The VLP platform has been deployed to develop vaccines for the recurring influenza pandemic [29,106,107,108]. The fast mass production of plant factory-derived VLPs could be a promising vaccine-manufacturing platform to cope with the COVID-19 pandemic.

For rapid vaccine production, Medicago has developed a proprietary high-throughput screening platform, VLPExpress, which enables testing of more than 200 different expression approaches per week. The platform has significantly accelerated the development of Medicago’s products and has enabled the production of a broad range of VLP-based vaccines as well as antibody candidates. Moreover, Medicago is developing an antigen-displaying platform to produce an effective VLP using an envelope and capsid protein that warrants incorporation and displays diverse antigens [109].

Medicago’s CoVLP vaccine is formulated with two types of adjuvants, ASO3 and CpG 1018. Vaccine formulation with an adjuvant is essential in designing a vaccine for a pandemic, since it facilitates a reduction in the quantity required for mass immunization by enhancing the efficacy of the vaccine, especially in the case of recombinant subunit vaccines that have low immunogenicity compared with inactivated and live-attenuated vaccines [79,110,111,112]. Therefore, to enhance the immunogenicity of the CoVLP vaccine by administration of an adjuvant, Medicago collaborated with global healthcare companies GlaxoSmithKline (GSK) and Dynavax Technologies for promising adjuvants. Dynavax’s CpG 1018 is a Toll-like receptor 9 antagonist adjuvant composed of a short (22-mer) oligonucleotide sequence containing CpG motifs [113]. CpG 1018 is effective and safe and is formulated with Dynavax’s hepatitis B vaccine, HEPLISAV-B^®^, approved by the USFDA [114,115]. The other adjuvant, AS03 from GSK, is composed of α-tocopherol, squalene, and polysorbate 80 in an oil-in-water emulsion and has been used in GSK’s Pandemrix, an H1N1 influenza vaccine [116,117].

Medicago’s first product, a quadrivalent VLP vaccine for seasonal influenza, completed the phase 3 clinical trial in 2020 and was the first plant-derived human vaccine that was well tolerated and provided substantial protection from influenza viruses in adults [48]. The company has more vaccine candidates in the production pipeline, including one against rotavirus and influenza; both are in phase 1 clinical trials [118]. Furthermore, it has produced a fully formulated HA VLP vaccine within 3 weeks of the release of the genetic sequence for the A/H1N1(A/California/04/09) strain [107]. These findings prove the capacity of the plant-based platform for rapid vaccine production, one of the most important aspects of vaccine development at the time of a pandemic. Medicago demonstrated its capacity for mass-scale production by producing 10 million doses of the monovalent H1N1 VLP vaccine in tobacco plants within 30 days in association with the DARPA Blue Angel program in 2012 [41]. The company expects to produce as many as 80 million doses from 2021 and over 1 billion doses of COVID-19 vaccines annually after the completion of a large-scale factory construction in Quebec in 2023 [118]. The success of the influenza VLP vaccine development has bolstered Medicago to progress the CoVLP vaccine to clinical trials in a short period.

### Clinical Trial Results for CoVLP

The provisional results of the phase 1 clinical trial of Medicago’s CoVLP vaccine were reported on November 10, 2020 [119]. In the trial, 3.76, 7.5, and 15 μg of CoVLP with or without ASO3 and CpG 1018 were intramuscularly injected into 18–55-year-olds at an interval of 21 days in a randomized, partially blinded clinical trial (NCT04450004) [120]. The immunogenicity results demonstrated that adjuvant formulations have a greater potential to improve humoral and cellular immune responses to the CoVLP vaccine compared with non-adjuvant formulations. All subjects receiving the adjuvanted vaccine developed neutralizing antibodies as the immune response after the second dose in all dose groups. All subjects receiving CoVLP formulated with ASO3 developed anti-spike IgG after a single dose. The antibody levels observed in vaccinated subjects were higher than those observed in the convalescent sera of people who recovered from COVID-19 [68]. The vaccine formulation with adjuvants significantly enhanced cellular Th1 immune responses in subjects receiving the 3.75 or 7.5 µg dose. However, no dose-dependent effect was observed for CoVLP formulated with CpG 1018, which might be the reason for its exclusion from the phase 2/3 clinical trial. No serious adverse effects were observed, except for mild-to-moderate short-term side effects [68,119].

With encouraging positive results, the CoVLP vaccine entered the phase 2/3 clinical trial (NCT04636697) on 12 November 2020 [121]. The trial was designed as a randomized, observer-blind, placebo-controlled study to evaluate the safety and immunogenicity of the recombinant CoVLP formulated with an adjuvant in adults aged 18–64 years and the elderly (aged 65+ years).

The trial employed a single dose, 3.75 µg, of CoVLP, and only one type of adjuvant, 0.5 mL of ASO3, and Medicago planned to enroll more than 30,000 participants aged 18 years and above. The interim report on the phase 2 portion of the clinical trial was released on May 18, 2021, reiterating the safety and immunogenicity of the CoVLP vaccine indicated by the phase 1 clinical trial. Double doses of 3.75 µg of CoVLP formulated with ASO3 significantly induced a humoral immune response in both age groups, although the response was greater in adults than in the elderly after a single dose [50]. The level of neutralizing antibodies induced by CoVLP plus ASO3 was 10 times higher than that observed in the convalescent sera of people who recovered from COVID-19. Vaccination with a single as well as a double dose of CoVLP plus ASO3 induced a significant cellular immune response via interferon gamma (IFN-γ) and IL-4 responses, which was relatively stronger in adults than in the elderly. Consistent with phase 1 trial results, adverse effects were mild to moderate and of a transient duration, such as injection site pain, muscle ache, and fatigue for 24 h to 3 days [50]. The CoVLP vaccine entered the phase 3 portion of the clinical trial on March 16, 2021. Furthermore, Medicago is developing antibodies against SARS-CoV-2 in collaboration with Laval University Canada [39].

The USFDA authorized fast-track designation for the CoVLP vaccine on 17 February 2021, and Health Canada initiated a review of the rolling submission of the CoVLP vaccine on 21 April 2021 [122,123]. This could certainly facilitate the speeding up of the vaccine’s availability for public immunization. The progress made in CoVLP vaccine development represents a milestone in plant-based vaccine development history for pandemics. This could contribute to the equal distribution of the vaccine worldwide, since the CoVLP vaccine can be stored at 2–8 °C, easing cold chain management with the existing infrastructure, a promising alternative to the currently available vaccines requiring ultracold storage temperatures.

## 4. KBP-201: A COVID-19 cVLP Vaccine in a Phase 1/2 Clinical Trial

US-based KBP, owned by the British American Tobacco (BAT) group, is one of the plant-based vaccine developers that has secured the second position in the race to develop a plant-based COVID-19 vaccine. The company’s COVID-19 vaccine, KBP-201, is currently in a phase 1/2 clinical trial (NCT04473690) [2,59]. KBP-201 is a protein subunit vaccine based on the SARS-CoV-2 receptor-binding domain (RBD) protein transiently expressed in *Nicotiana benthamiana* [2,52].

KBP also selected the S protein of SARS-CoV-2 for vaccine design but procured only partial sequences—that is, the RBD. The RBD is excessively exploited in COVID-19 vaccine development for its major role in virus cell entry and high immunogenicity [90,91,92]. The role of the S protein in viral cell entry is largely contributed by the RBD. The S protein is composed of two functional subunits, a receptor-binding protein, S1, constituting RBD, and a membrane fusion protein, S2. Three copies of both S1 and S2 are arranged to form a trimer with a stalk and a head, with S1 positioned as the head on the top of a stalk of S2. Viral cell entry initiates upon the attachment of S1 to the cell via the binding of the flexible RBD at its standing position with ACE2 [92,93,124,125]. Therefore, an RBD vaccine can protect the immunized organism against SARS-CoV-2 infection at the very beginning, during viral cell entry. Additionally, similar to the S protein, most of the SARS-CoV-2-neutralizing antibodies from COVID-19-recovered patients are against the RBD protein [94,95,96], providing additional rationale for selecting the RBD for COVID-19 vaccine design.

KBP uses a unique approach to KBP-201 vaccine production, in which antigens of SARS-CoV-2 and modified tobacco mosaic virus (TMV) are separately expressed in the tobacco plant and the plant-derived RBD and TMV are chemically assembled to produce the vaccine after purification [52,126]. The vaccine produced by the assembly of antigen and virus is known as a chimeric VLP (cVLP) vaccine, which resembles the VLP vaccine, since, in both vaccines, a self-assembled virus creates a particle structure to provide a scaffold for displaying the target antigen. However, the cVLP vaccine is composed of an antigen-unrelated virus, which means that it displays heterologous antigens, while the VLP vaccine displays its own antigens [127].

The ability of plant viruses to form VLPs by the self-assembly of single or multiple proteins makes them ideal carrier proteins for cVLPs that can be conjugated with a foreign antigen to develop a potential vaccine [128,129]. The application of plant viruses, such as TMV, as a VLP carrier protein offers two advantages: the vaccines are safe since plant viruses are non-infectious to humans, in contrast to mammalian-origin viruses, and they can be easily produced in plants with genetically fused antigens or plant viruses. The unique combination of a plant-derived vaccine with TMV has the potential to be stable at room temperature. The first plant-virus-derived cVLP vaccine against poliovirus was developed using TMV. Afterwards, numerous plant-virus-based vaccines have been developed and evaluated for their efficacies [36,71,130].

KBP used *N. benthamiana* to express the RBD antigen and TMV for the rapid production of KBP-201 [131,132]. The company developed an innovative technology based on a fast-growing tobacco plant that has superior potential to conventional vaccine production technology: speed, cost-effectiveness, scalability, robustness, and flexibility. KBP owns an automated plant growth facility that can grow 3 million tobacco plants in a climate-controlled environment, which can be optimized to produce a protein of interest within 6–8 weeks. The adequately available plant growth facility ensures the mass production of KBP-102 immediately after completion of the clinical trial.

KBP’s experiences with plant-based pharmaceutical production, especially during the 2014 Ebola outbreak, helped to accelerate COVID-19 vaccine development. During the 2014 Ebola outbreak, KBP produced three Ebola monoclonal antibodies to prepare enough cocktails to treat six affected persons at a dose of 50 mg/kg body weight [63]. The product was developed by the public health agency of Canada’s National Microbiology Laboratory, the Army Medical Research Institute of Infectious Disease, USA, and Zmapp Biopharmaceuticals and was manufactured by KBP. KBP has two vaccine candidates in the pipeline, the quadrivalent seasonal influenza vaccine KBP-V001 (NCT04439695), which entered the phase 1 clinical trial in June 2020, and an influenza vaccine, which is in the preclinical development stage [133].

The phase 1/2 clinical trial of KBP-201 started on December 30, 2020. The trial is designed as an observer-blinded, randomized, placebo-controlled, parallel-group study to evaluate the safety and immunogenicity of KBP-201 plus CpG (adjuvant) in healthy SARS-CoV-2 seronegative adults in two age groups: A (18–49 years) and B (50–85 years) [59]. To evaluate dose-dependent immunogenicity, each age group is divided into two subgroups, low dose and high dose, for the administration of 15 μg of KBP-201 plus 0.5 mg of CpG and 45 μg of KBP-201 plus 0.5 mg of CpG. The study subjects are randomized in a 1:1:1 ratio to receive two doses of KBP-201 by intramuscular injection at an interval of 21 days. The trial is recruiting 180 participants, and the results are yet to be published.

## 5. COVID-19 Vaccines in the Preclinical Stage

The WHO has listed 184 different COVID-19 vaccines in preclinical studies, including three plant-based vaccines [2]. In addition, plant biologists and biotech companies worldwide are developing plant-based COVID-19 vaccines, which are in the discovery and preclinical phases and are yet to be listed in the WHO vaccine list.

G+FLAS Life Sciences, Korea, transiently expressed antigens from the SARS-CoV-2 S protein and RBD in *N. benthamiana* to develop a COVID-19 protein subunit vaccine and investigated the immunogenicity of the tobacco-produced antigen in mice [57]. To produce an efficient vaccine, the company used the glycoengineered tobacco plant to express antigens, since glycosylation of antigens affects the antigenicity and efficacy of the vaccine [19,134,135,136]. The S protein contains 22 N-linked glycosylation sites, indicating that differently glycosylated antigens could result in a vaccine with enhanced efficacy [137]. The glycosylation process in plants and mammals is different; thus, the glycotrait of the recombinant vaccine manufactured in two different host cells may vary, affecting its efficacy. For instance, the anti-CD20-hIL-2 immunocytokine expressed in the tobacco plant devoid of plant-specific xylose/fucose N-glycosylation shows better biological activity compared with immunocytokines obtained from animal cells, as evidenced by enhanced antibody-dependent cellular cytotoxicity and Fc-c receptor binding [138]. The preclinical study demonstrated that the G+FLAS RBD antigen with modified glycosylation can elicit a humoral immune response with the induction of highly neutralizing antibodies in mice which protected the Vero E6 cell from SARS-CoV-2 virus infection [139]. The company is expected to manufacture approximately 20,000 doses of vaccine per production cycle in the currently available plant growth facility [140].

iBio, a biotech company in the US, produced COVID-19 vaccine candidates IBIO-200, IBIO-201, and IBIO-202 by adopting two different production platforms, VLP and protein subunit vaccines, respectively [51]. In association with Texas A&M University System (TAMUS), iBio developed two forms of SARS-CoV-2 VLPs, glycosylated IBIO-200 and nonglycosylated IBO-200 [141]. Both vaccine candidates, in combination with different adjuvants, were tested in mice to evaluate their immune responses, including the induction of neutralizing antibodies against SARS-CoV-2. The preclinical study revealed promising results with IBIO-201, and a subsequent in vivo toxicology study commenced. IBIO-201 is a carrier protein-conjugated subunit vaccine generated by the fusion of the S protein with LicKM, the company’s patented booster molecule [51]. Carrier molecules are often fused with the target antigen to enhance the expression, stability, and immunogenicity of the vaccine. LicKM is an engineered thermostable lichenase enzyme originally from *Clostridium thermocellum* [142]. The plant-produced LicKM’s conjugation with the target antigen demonstrated enhanced expression, solubility, stability, and immunogenicity of the vaccine [142,143,144]. The safety and protective efficacy of the LiCKM-conjugated vaccine have been evaluated against pneumonic plague in nonhuman primates [69,145]. The company expects an increase in the potency of subunit vaccines as well as the durability of the immune response by LicKM [146]. The fusion antigen thermostability conferred by LicKM can support the easy and cost-effective recovery of IBIO-201 during purification, since the target antigen can be separated from other proteins by heat treatment [142].

Gram quantities of high-quality IBIO-201 antigens from *N. benthamiana* can be produced rapidly using iBio’s FastPharming system, which combines an automated hydroponic system, vertical farming, and glycan engineering technology. The FastPharming system has an accelerating feature for the development of candidate biopharmaceuticals into a clinical product. It requires 10 months for the generation of master cell banking and 14 days for manufacturing clinical products, while the mammalian cell culture system requires over 12 months and 40 days, respectively [51]. The company’s glycoengineering technology can be used to produce antigens with various antigenicities to select the most effective candidate.

On 6 May 2021, iBio reported the completion of the toxicology study of IBIO-201, reporting no adverse effect at both low and high doses, which enabled the investigational new drug submission process. In addition to COVID-19 vaccines, iBio is developing a COVID-19 therapeutic ACE-Fc, ACE2 fused with the Fc region of human IgG1, in collaboration with Planet Biotechnology [147]. The company has developed the veterinary vaccine IBIO-400 for classical swine fever and IBIO-11 for fibrotic disease [51].

Baiya Phytopharm, Thailand, developed six different prototypes for COVID-19 vaccines in *N. benthamiana*. Of the six vaccine candidates, Baiya SARS-CoV Vax 1 was successfully tested in mice and monkeys [60]. Preclinical results demonstrated effective stimulation of neutralizing antibodies in the vaccinated animals after two doses. Based on these positive results, the company is assessing the toxicology and side effects of the vaccine. It plans to submit a proposal to the country’s authorities for human trials if the candidate vaccines show efficacy and safety with low or no toxicity. The company expects to start clinical trials by the middle of 2021.

Academia from different countries also participated in the development of a plant-based COVID-19 vaccine. Chulalongkorn University, Thailand, in collaboration with Baiya Phytopharm, transiently expressed the RBD protein in *N. benthamiana*, and the plant-produced RBD was found to be efficiently bound to ACE2 [54]. The team used the plant-derived RBD to develop a COVID-19 detection reagent, the Baiya Rapid COVID-19 IgG/IgM test kit, using a lateral flow immunoassay (LFIA) strip. The LFIA diagnostic kit demonstrated a sensitivity and specificity of 94.1% and 98%, respectively, for IgG and IgM antibodies against SARS-CoV-2 in human sera [49]. LFIA can produce test results in 15 min and can be visually analyzed. The team applied to Thailand’s FDA for medical device approval in April 2020. The progress accomplished by the group has extended the potential of plant-based antigen production for rapid and cost-effective diagnostic kit development to combat the COVID-19 pandemic. The team has also demonstrated its capacity in plant-based heterologous protein expression and the effort toward COVID-19 by producing ACE-Fc and SARS-CoV-2 neutralizing antibodies B38 and H4 [53,55].

In collaboration with Cape Biopharm, the University of Cape Town expressed spike S1 and RBD antigens in *N. benthamiana*. Using these proteins, the group established an indirect enzyme-linked immunosorbent assay (ELISA) for serological tests to detect SARS-CoV-2 antibodies. The ELISA developed using plant-derived S1 and RBD antigens demonstrated sensitivity and specificity comparable to the commercial ELISA kit [56].

## 6. Second-Generation COVID-19 Vaccines in the Preclinical Stage

The constant evolution of SARS-CoV-2 through mutation, a characteristic feature of the RNA virus, has led to the emergence of more than 4000 variants globally [148]. Some of the mutations have attracted extensive attention since they have potential for increased transmissibility, increased virulence, and immune evasion, leading to reduced effectiveness of vaccines. The reduced efficacy of existing vaccines, including NVX-CoV2373, Novavax, Ad26.COV2.S, Johnson and Johnson; ChAdOx1-S, AstraZeneca, BNT162b2, Pfizer; and mRNA-1273, Moderna, against the South African variant, B.1.351, has been reported [148]. Hence, considering that most of the currently available vaccines are based on the S protein, while SARS-CoV-2 mutations are appearing on the S gene sequence, there is a critical need to develop new vaccines targeting antigens other than the S protein [148,149,150,151,152]. Furthermore, designing multiepitope and multivalent vaccines could be an effective approach to protecting against unpredictably modifying SARS-CoV-2. The nucleocapsid (N) protein could be a potential target antigen since the N protein is highly immunogenic and more conserved among SARS-CoV-2 variants and other coronaviruses [153,154,155]. A vaccine based on an N protein antigen with a conserved sequence may provide broader protection against a wide range of SARS-CoV-2 variants [61]. In light of variant emergence, researchers working on plant-based vaccine production have initiated efforts for the development of second-generation vaccines.

Researchers from Akdeniz University, Turkey, expressed the S1 protein, the RBD, and the N protein transiently in the tobacco plant and studied the immunogenicity [156]. The group, for the first time, produced N antigens alone and in combination with the RBD and S1 domain in the plant, whereas most plant-based vaccine developers have targeted the RBD, S1 protein, and S protein as antigens. In addition, the group produced in vivo deglycosylated variants of these antigens by co-expression of endoglycosidase (EndoH). The plant-derived RBD and S1 antigens specifically bound to ACE2, the binding being stronger in deglycosylated versions. All the antigens (N protein, S1 protein, and the RBD) elicited high-titer antibodies in mice. Immunization with two antigens, N protein plus RBD, elicited high-titer antibodies compared with the RBD and N protein alone, and one dose of the N protein plus RBD was enough to produce almost the same level of antibodies as observed after two doses of the RBD and N protein alone. The N protein-based vaccine approach can be applied for the development of next-generation COVID-19 vaccines to complement the existing S protein-based vaccines, as demanded by the global emergence of SARS-CoV-2 mutations and the reduced efficacy of existing vaccines against the variants. The enhanced immunogenicity of the cocktail administration of the RBD plus the N protein indicates that immunization with more than one antigen can protect without a booster dose, and a combination of different target antigens can protect from more than one variant.

On 6 May 2021, iBio announced a new subunit vaccine candidate, IBIO-202, designed to target the N protein as a second-generation COVID-19 vaccine aiming to resolve the vaccine efficacy issues generated by the emerging variants. iBio has proceeded with a preclinical study of IBIO-202 to identify an effective adjuvant and evaluate the efficacy of the vaccine. Furthermore, iBio is examining the potential of multisubunit vaccine candidates by targeting more than one antigen to enhance the protective efficacy of the vaccine against emerging variants [146].

## 7. Conclusions and Future Perspectives

Immunization is the most effective measure to prevent infectious diseases; hence, it has received the attention of the world in an effort to combat the COVID-19 pandemic. The tireless efforts of the scientific community and health workers led to the development of COVID-19 vaccines within a year of the first report of the outbreak. The plant-based vaccine production system has also been exploited to develop COVID-19 vaccines, and some of the candidate vaccines are in clinical trials. This review described the ongoing efforts in the development of plant-based COVID-19 vaccines, the progress so far, recent results of clinical trials, and strategies and technologies used for vaccine production. Plant-based vaccine candidates are mostly based on the S protein of SARS-CoV-2, but a new antigen target, the N protein, is also being used by some groups. VLP, cVLP, protein subunit, and carrier molecule-fused protein subunit vaccines against COVID-19 have been developed in the tobacco plant. In addition to the vaccines, plant-based antigens are used for antigen-based diagnostic reagent development, extending the scope of the plant-based production system in response to COVID-19. The rapid mass production of a vaccine for clinical trials can be achieved with the application of a transient expression system based on viral vectors in the fast-growing and high-biomass-producing *N. benthamiana* as a host plant.

The progress of Medicago’s CoVLP vaccine into phase 3 and KBP’s KBP-201 into phase 1 clinical trials has manifested the plant as an effective production platform for rapid, robust, safe, and economic vaccine production to respond to the COVID-19 pandemic. The productive achievement in COVID-19 vaccine development can be regarded as a milestone in the history of plant-based biopharmaceutical development. Moreover, it can encourage authorities to accept the plant-based technique as one of the vaccine production systems and to prioritize it as part of the major plan to combat future outbreaks. The progress accomplished in COVID-19 vaccine development in the past 1.5 years emphasizes the importance of collaboration among academia and industries from multidisciplinary fields and information sharing among scientific communities for the rapid progression of vaccine development.

At the time of writing this article, 212 countries had administered 2.59 billion doses of the COVID-19 vaccines by 19 June 2021 [157,158]. Most of the vaccines require double doses for effective protection. Considering that the total population of the world is 7.9 billion, the world still needs the mass production of billions of doses. The plant-based production system may fulfill a certain percentage of the global vaccine need. However, there is a huge gap in vaccine distribution between developed and developing countries. Countries with a low economy are keeping track of the WHO Covax program for vaccination of their people. In addition, the currently available mRNA vaccine requires an ultracold storage system, which hinders the developing countries’ accessibility to vaccines. Plant-based vaccine technology potentially overcomes both of these obstacles faced by developing countries by eliminating the necessity of ultralow-temperature storage systems and mass production at a low price. In other words, plant-based vaccine technology can facilitate the equitable distribution of vaccines globally.

Several new variants of SARS-CoV-2 are emerging around the world, and currently available vaccines are less responsive to these new variants. The reason may be the high rate of mutations in the S protein, as most of the vaccines are based on it. In this scenario, a novel vaccine targeting a protein other than the S protein, such as the N protein, could be a potential alternative, since the N protein is highly immunogenic and more conserved, suggesting less vulnerability to mutation. Immunization with more than one antigen may provide satisfactory protection, eliminating the need for a booster dose. People can be immunized by a combination of different target antigens to protect against multiple strains of coronavirus. A robust production system, selection of a broad range of target antigens, and the development of multiepitope and multivalent vaccines integrated with plants could be effective approaches to overcoming the challenges created by SARS-CoV-2 mutational variants.

## Figures and Tables

**Table 1:** Plant-based coronavirus disease 2019 (COVID-19) vaccine candidates.

Vaccine	Vaccine Platform	Target Antigen	Development Phase	Developer	Country	References
CoVLP	VLP	S protein	Phase 2/3	Medicago	Canada	[50,58]
KBP-201	cVLP	RBD	Phase 1/2	Kentucky Bioprocessing	United State	[59]
IBIO-200	VLP	S protein	preclinical	iBio	United State	[51]
IBIO-201	Conjugated protein subunit	S protein	preclinical	iBio	United State	[51]
IBIO-202	Protein subunit	N protein	preclinical	iBio	United State	[51]
RBD	Protein subunit	RBD	preclinical	G+FLAS Life Sciences	South Korea	[57]
Baiya SARS-CoV Vax 1	Protein subunit	NM *	preclinical	Baiya phytopharm	Thailand	[60]
S1 protein	Protein subunit	S1 protein	preclinical	Akdeniz University	Turkey	[61]
RBD	Protein subunit	RBD	preclinical	Akdeniz University	Turkey	[61]
N protein	Protein subunit	N protein	preclinical	Akdeniz University	Turkey	[61]

* Not mentioned.

**Table 2:** Plant-based vaccines in clinical trials.

Disease	Pathogen	Antigen	Host Plant	Expression System	Route of Administration	Clinical Phase	References
Seasonal Influenza	A/H1N1, A/H3N2, B/Brisbane, B/Phuket	HA Quadrivalent	*Nicotiana benthamiana*	Transient VLP	Intramuscular	Phase 3 completed	[48,66]
COVID-19	SARS-CoV-2	Spike protein	*Nicotiana benthamiana*	Transient VLP	Intramuscular	Phase 3	[50,58]
COVID-19	SARS-CoV-2	RBD	*Nicotiana benthamiana*	Transient cVLP	Intramuscular	Phase 1/2	[59]
Influenza	H5N1	HA (H5)	*Nicotiana benthamiana*	Transient	Intramuscular	Phase 2 completed	[79]
Influenza	H5N1	HA	*Nicotiana benthamiana*	Transient	Intramuscular	Phase 1 completed	[73]
Influenza	H1N1 virus	HA	*Nicotiana benthamiana*	Transient	Intramuscular	Phase 1 completed	[70]
Malaria	Plasmodium falciparum	Pfs25 VLP	*Nicotiana benthamiana*	Transient cVLP	Intramuscular	Phase 1 completed	[71,72]
Influenza	H7N9	HA (H7)	*Nicotiana benthamiana*	Transient	Intramuscular	Phase 1	[27,74]
Cholera	Vibrio Cholera	CTB	Rice	Transgenic	Edible	Phase 1	[75]
Hepatitis B	HBV	HBsAg	Potato	Transgenic	Edible	Phase 1	[76]
Hepatitis B	HBV	HBsAg	Lettuce	Transgenic	Edible	Phase 1	[35]
Rabies	Rabies virus	G protein	Spinach	Transient	Oral	Phase 1	[36]
Gastroenteritis	Norwalk virus	Capsid protein	Potato	Transgenic	Oral	Phase 1	[77]
Gastroenteritis	Norwalk virus	NM *	*Nicotiana benthamiana*	Transient VLP	NA	Phase 1	[78]
Anthrax	*Bacillus anthracis*	Protective antigen	*Nicotiana benthamiana*	Transient	N	Phase I	[64]
Gastroenteritis	Rotavirus	NM *	*Nicotiana benthamiana*	Transient VLP	NA	Phase 1	[65]

* Not mentioned; COVID-19, coronavirus disease 2019.

**Table 3:** Plant-based vaccines for epidemics in preclinical study.

Disease	Antigen	Host Plant	Expression System	Efficacy in Preclinical Study	References
Plague	F1 and V	Tomato	Transgenic	50% protection in mice	[80]
Plague	F1 and V	*Nicotiana benthamiana*	Transient	88% protection in monkeys	[69]
HIV/AIDS Pandemic	HIV multi proteins	*Nicotiana benthamiana*	Transplantomic	Induced humoral and cellular immune response in mice.	[82]
HIV/AIDS Pandemic	p24	*Arabidopsis thaliana*	Transgenic	Induced humoral immune response in mice.	[86]
Dengue fever	SP and NSPs	*Nicotiana benthamiana*	Transient VLP	Induced humoral immune response in mice.	[89]
Dengue fever	EDIII-1-4 tetravalent antigen	*Lettuce*	Transplantomic	Induced specific antibodies in rabbits.	[88]
Yellow fever	Envelop protein	*Nicotiana benthamiana*	Transient	77% protection in mice and induced cellular and humoral immune response in monkey.	[83]
Yellow fever	Envelop protein	*Nicotiana benthamiana*	Transient	100% protection in mice and induced cellular and humoral immune response in monkey.	[84]
Ebola	Glycoprotein (GP1)	*Nicotiana benthamiana*	Transient	Induced anti-Ebola antibodies in mice.	[87]

HIV/AIDS, human immunodeficiency virus/acquired immunodeficiency disease.

## Data Availability

The data are included in the article.
